# Patient Assessment Chronic Illness Care (PACIC) and its associations with quality of life among Swiss patients with systemic sclerosis: a mixed methods study

**DOI:** 10.1186/s13023-022-02604-2

**Published:** 2023-01-09

**Authors:** Agnes Kocher, Michael Simon, Andrew A. Dwyer, Catherine Blatter, Jasmina Bogdanovic, Patrizia Künzler-Heule, Peter M. Villiger, Diana Dan, Oliver Distler, Ulrich A. Walker, Dunja Nicca

**Affiliations:** 1 Department Public Health (DPH), Institute of Nursing Science (INS), Faculty of Medicine, University of Basel, Bernoullistrasse 28, 4056 Basel, Switzerland; 2 Department of Rheumatology and Immunology, Inselspital, Bern University Hospital, University of Bern, Bern, Switzerland; 3 Department of Nursing, Inselspital, Bern University Hospital, University of Bern, Bern, Switzerland; 4 Boston College, Connell School of Nursing, Chestnut Hill, MA USA; 5 Center for Nursing Research, Massachusetts General Hospital Munn, Boston, MA USA; 6 Department of Gastroenterology/Hepatology, Cantonal Hospital St. Gallen, St. Gallen, Switzerland; 7 Department of Nursing, Cantonal Hospital St. Gallen, St. Gallen, Switzerland; 8 Medical Center Monbijou, 3011 Bern, Switzerland; 9 Service of Rheumatology, Lausanne University Hospital, University of Lausanne, Lausanne, Switzerland; 10 Department of Rheumatology, University Hospital Zurich, University of Zurich, Zurich, Switzerland; 11 Department of Rheumatology, University Hospital Basel, Basel, Switzerland; 12 Department of Global and Public Health, Institute for Epidemiology, Biostatistics and Prevention, University of Zurich, Zurich, Switzerland

**Keywords:** Health-related quality of life, Health services research, Nursing, Outcome and process assessment, Patient-centered care, Patient-reported outcome measures, Rare diseases, Rheumatology, Scleroderma, Systemic sclerosis

## Abstract

**Background:**

The Chronic Care Model (CCM) is a longstanding and widely adopted model guiding chronic illness management. Little is known about how CCM elements are implemented in rare disease care or how patients’ care experiences relate to health-related quality of life (HRQoL). We engaged patients living with systemic sclerosis (SSc) to assess current care according to the CCM from the patient perspective and their HRQoL.

**Methods:**

We employed an explanatory sequential mixed methods design. First, we conducted a cross-sectional quantitative survey (n = 101) using the Patient Assessment of Chronic Illness Care (PACIC) and Systemic Sclerosis Quality of Life (SScQoL) questionnaires. Next, we used data from individual patient interviews (n = 4) and one patient focus group (n = 4) to further explore care experiences of people living with SSc with a focus on the PACIC dimensions.

**Results:**

The mean overall PACIC score was 3.0/5.0 (95% CI 2.8–3.2, n = 100), indicating care was ‘never’ to ‘generally not’ aligned with the CCM. Lowest PACIC subscale scores related to ‘goal setting/tailoring’ (mean = 2.5, 95% CI 2.2–2.7) and ‘problem solving/contextual counselling’ (mean = 2.9, 95% CI 2.7–3.2). No significant correlations were identified between the mean PACIC and SScQoL scores. Interviews revealed patients frequently encounter major shortcomings in care including ‘experiencing organized care with limited participation’, ‘not knowing which strategies are effective or harmful’ and ‘feeling left alone with disease and psychosocial consequences’. Patients often responded to challenges by ‘dealing with the illness in tailored measure’, ‘taking over complex coordination of care’ and ‘relying on an accessible and trustworthy team’.

**Conclusions:**

The low PACIC mean overall score is comparable to findings in patients with common chronic diseases. Key elements of the CCM have yet to be systematically implemented in Swiss SSc management. Identified gaps in care related to lack of shared decision-making, goal-setting and individual counselling-aspects that are essential for supporting patient self-management skills. Furthermore, there appears to be a lack of complex care coordination tailored to individual patient needs.

**Supplementary Information:**

The online version contains supplementary material available at 10.1186/s13023-022-02604-2.

## Background

Systemic sclerosis (SSc) is a rare multisystemic, autoimmune connective‐tissue disease characterized by a chronic and frequently progressive disease course. Approximately 20 in 100,000 adults are affected [1, 2]. Variability in disease severity, progression, and organ involvement challenge timely diagnosis and effective disease management contributing to high mortality [1, 3]. Approximately 75% of patients develop organ involvement within the first five years of diagnosis and early manifestations including skin fibrosis (75%), gastrointestinal symptoms (71%), lung involvement (65%), digital ulcers (34%) and cardiac involvement (32%) [3]. Except for haematopoietic stem cell transplantation for patients with rapidly progressive dcSSc and a high risk of organ failure in an early disease stage, treatments modifying the overall disease course are currently not available [4, 5]. Thus, medical management must be tailored to individual organ sequelae and disease progression, i.e., regular multidisciplinary consultations to identify organ involvement early as well as pharmacological and non-pharmacological interventions to decrease/slow disease progression and reduce organ damage [4].

At the same time, interventions should focus on improving health-related quality of life (HRQoL) of people living with SSc [6]. Over the disease trajectory, patients experience multiple physical and psychosocial problems including fatigue, hand stiffness, Raynaud’s phenomenon, digital ulcers, shortness of breath, pain, gastrointestinal symptoms, work disability, depression, anxiety (e.g., fear of disease progression), and dissatisfaction with body image [6–10]. Numerous studies report severely impaired physical and psychological HRQoL in SSc [10–13]. Importantly, the heterogeneous disease presentation and the symptom burden of patients living with SSc necessitate a chronic care approach including competent, coordinated, multidisciplinary collaboration as well as self-management support targeting individual patient needs [14–17]. However, prevailing models of SSc care mainly focus on acute health problems and often lack an integrated approach that addresses the complex care needs of patients [18, 19].

The Chronic Care Model (CCM) is a longstanding and widely adopted model that includes electronic health (eHealth) approaches to guide chronic illness management [19–21]. The model aims to improve health outcomes through effective and productive interactions between prepared, proactive practice teams and informed, activated patients. The CCM focuses on the six core elements: community resources, health system, self-management support, delivery-system design (e.g., continuity of care), decision support, and clinical information systems. A significant body of literature supports that incorporating CCM elements (e.g. self-management support, clinical decision support) into care is associated with better clinical outcomes including reduced health service use, fewer emergency department visits and lower healthcare costs [22–24]. The Patient Assessment of Chronic Illness Care (PACIC) is a validated tool to assess implementation of the CCM from the patient perspective [25]. Notably, several studies have shown that perceived level of chronic illness management (as measured by the PACIC) is positively correlated with patient outcomes [26–28]. For example, in diabetes, higher PACIC scores are associated with improved markers of glycemic control, self-management activities, physical activity and diminished distress [29, 30]. In transplant patients, higher perceived levels of chronic illness management are positively associated with treatment satisfaction and trust in the transplantation team [31].

In the rare disease space, few care models incorporate elements of the CCM [32–34]. On average, rare disease patients reported PACIC score of 2.5 (on a 5-point Likert scale ranging from 1 = ‘never’ to 5 = ‘always’), suggesting poorer healthcare experiences compared to reports in patients with common chronic conditions (range 1.7–4.2/5.0) [26, 35–37]. In relation to SSc, the association between healthcare provision and rare disease patient outcomes (e.g., HRQoL) has remained unexplored. However, the PACIC is a disease-agnostic instrument that may not address specific challenges of rare disease care (e.g., lack of treatment options and specialized healthcare) or specific patient needs regarding heterogeneity/severity of SSc. Accordingly, SSc patient experiences of chronic care in relation to the PACIC dimensions may demand further inquiry using qualitative methods.

To date, there is paucity of evidence on how CCM elements are implemented in SSc management and how patients’ care experiences relate to HRQoL. The *MANagement Of Systemic Sclerosis (MANOSS)* project aims to fill existing gaps in SSc care by developing an eHealth-enhanced rare disease chronic care model for SSc patients in Switzerland [38]. As part of the *MANOSS* project, this mixed methods study aimed to describe the current state of SSc chronic illness care and HRQoL from the patient perspective to inform the development of an integrated model of care for SSc. The quantitative phase evaluated the level of chronic care across the five dimensions measured by the PACIC scale and quality of life measured by the Systemic Sclerosis Quality of Life Questionnaire (SScQoL). The subsequent qualitative phase aimed to explain care experiences of people living with SSc with a focus on the PACIC dimensions.

## Methods

### Study design

**Fig. 1:**
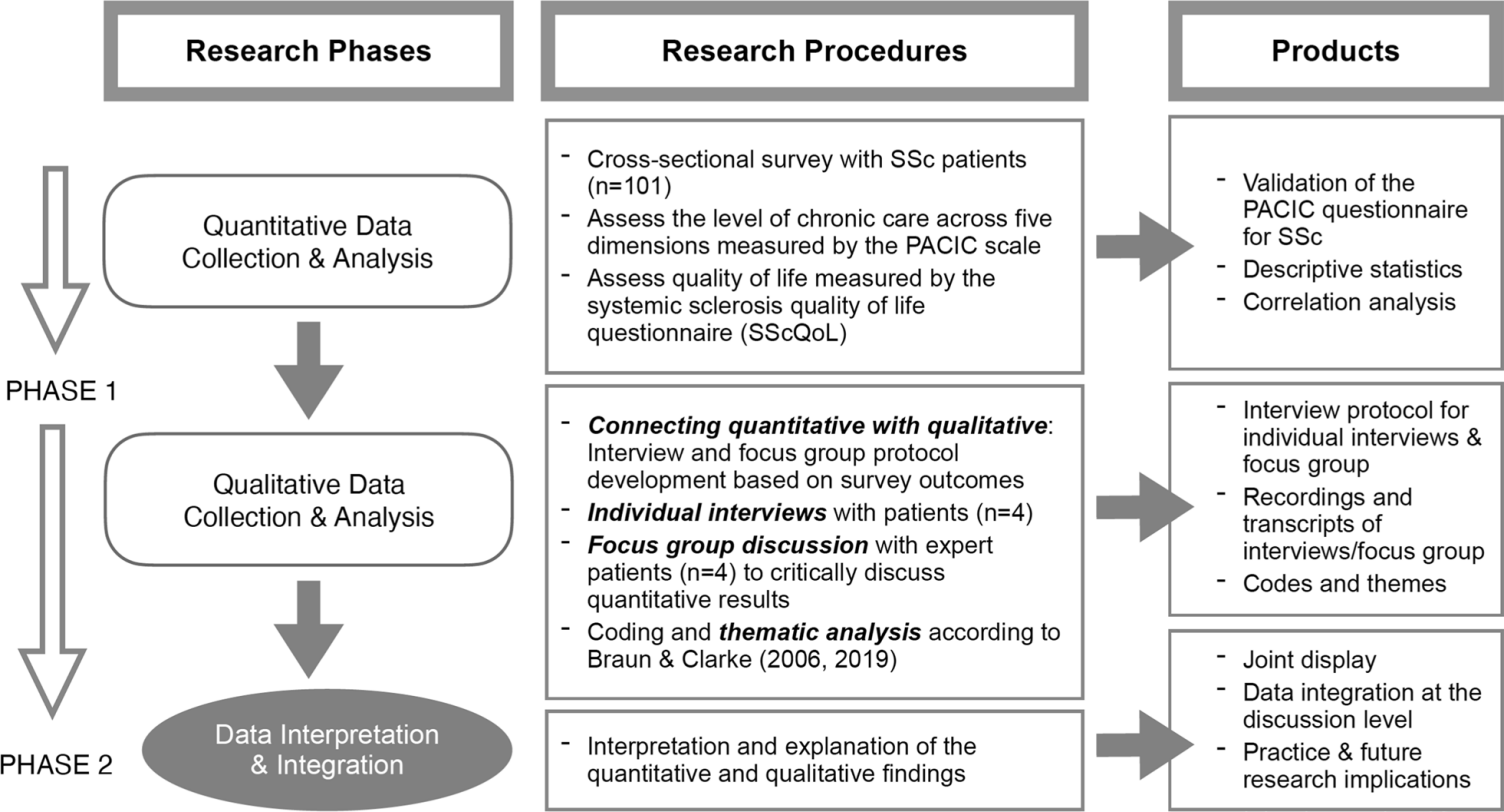
Study diagram for the explanatory, sequential mixed methods design

The study employed an explanatory, sequential, mixed methods design [38, 39]. Briefly, we first conducted a quantitative cross-sectional survey of Swiss SSc patients (see Fig. 1). Quantitative analyses informed subsequent qualitative interviews. For qualitative interviews, we used a purposefully selected sub-sample of patients based on the maximum variation of PACIC/HRQoL score to better understand and explain quantitative findings. Ethical approval was obtained for the overall *MANOSS* project by the responsible Swiss ethics committee (EKNZ 2018‐01206).

### Quantitative data collection and analysis

#### Sample and setting

For the quantitative survey, we recruited a convenience sample of 101 adult patients (> 18 years) spanning a range of SSc disease severity and experiences. We recruited German- and French-speaking participants from all Swiss University Hospitals, rheumatology outpatient clinics, and the Swiss scleroderma patients’ association (www.sclerodermie.ch) [38].

#### Variables and measurement

Patients participating in the *MANOSS* cross-sectional survey (March–August 2019) completed three survey instruments (paper or web-based format) [38]. We used the validated 20-item PACIC instrument to measure care alignment with CCM. The PACIC includes five subscales addressing specific domains: (1) patient activation; (2) delivery system design/decision support; (3) goal setting/tailoring; (4) problem solving/contextual counselling; and (5) follow-up/coordination [25]. Patients rated care received from their healthcare team (e.g., physicians, nurses, physiotherapists, occupational therapists, social workers) during the past 6-months using a 5-point Likert scale (1 = ‘never’ to 5 = ‘always’). Total and subscale scores (i.e., summed items completed within that scale) are averaged across items. The 20-item PACIC demonstrates reasonable validity and reliability, including high internal consistency (α = 0.93), in patients with chronic conditions across many languages and countries when using a single-dimension structure [25, 37, 40]. However, several studies have revealed high inter-correlations between PACIC subscales and ‘lack of fit’ using the 5-dimension structure—suggesting that subscales may not always be appropriate [37, 40, 41]. Because PACIC has not been used in the context of SSc, we used the Mokken model to test the construct validity of the PACIC scale and its subscales [42]. Additional details on our validation of the PACIC-15 for SSc are provided in Additional file 1.

We used the revised German and French 29-item Systemic Sclerosis Quality of Life (SScQoL) questionnaire to measure HRQoL [43, 44]. The revised German SScQoL employs a 4-point response structure (‘Always’, ‘Usually’, ‘Sometimes’, ‘Never’) and is a valid, reliable measure [44]. The response structure of the French version was adapted according to the German version to ensure interoperable responses for the *MANOSS* survey (German: α = 0.97, French: α = 0.91) [44, 45]. To calculate the overall SScQoL sum score, responses are dichotomized (‘Always’ = 1, ‘Usually’ = 1, ‘Sometimes’ = 1, ‘Never’ = 0) and summed. Higher values indicate lower HRQoL [44].

We assessed self-reported comorbidities using the 12‐item Self‐Administered Comorbidity Questionnaire (SCQ) that is moderately to strongly correlated with a standard medical record-based comorbidity measure (i.e., Charlson Index) [46]. Patients with SSc often struggle to distinguish between disease-related organ involvement and comorbidities unrelated to SSc. Thus, we used the SCQ to comprehensively assess self-reported comorbidity (i.e., co-occurring diseases in an individual) [47, 48]. Participants provided sociodemographic data (sex, age, education, employment status), disease information (subset: lSSc, dSSc, Overlap syndrome or unknown) and disease duration.

#### Quantitative data analysis

Quantitative data are reported using descriptive statistics (frequencies/percentages or means/medians with 95% confidence intervals and interquartile ranges) (R, Version 3.6.3, and DescTools-package) [49]. To compare PACIC-15 mean scores between groups (e.g., sex, age groups, education, comorbidities), we computed standardized mean differences (SMD)—which are identical to Cohen’s *d* (tableone-package for R) [50]. Compared to *p* values, SMD is more appropriate for calculating effect size estimates in small, uneven datasets—such as the ones analysed in this study [51]. A SMD ≥ 0.2, ≥ 0.5 and ≥ 0.8 depict small, medium and large differences between groups respectively. We calculated 95% confidence intervals (CIs) for means to facilitate comparison between ratings. Differences between groups were defined as means with distinct, non-overlapping CIs. Correlation analysis (pearson’s *r)* was computed to calculate associations between PACIC and SScQoL levels and visualized using the corrplot-package in R [52].

### Qualitative data collection and analysis

#### Sample and setting

To further explore the association between HRQoL and perception of chronic care, we used data from individual patient interviews (n = 4) and one patient focus group (n = 4), that were conducted within the larger qualitative *MANOSS* study (i.e., n = 14 individual interview and n = 17 focus group participants). Individual interview participants were purposefully selected from the quantitative *MANOSS* study sample according to patients’ PACIC and SScQoL scores (Table 1). For the focus group, we contacted patients with experience living with SSc (i.e., disease duration > 10 years) from the Swiss Scleroderma Association and the quantitative study sample. Participant background/profession (i.e., medical, scientific) was identified in the discussion round of the study.

**Table 1:** Interview subsample (n = 4) selected according to SScQoL and PACIC mean values

**Low quality of life (i.e., high SScQoL score)**	**High quality of life (i.e., low SScQoL score)**
**Low chronic care (i.e., low PACIC score)**	**Low chronic care (i.e., low PACIC score)**
*Patient 1*	*Patient 2*
SScQoL: 26/29	SScQoL: 5/29
PACIC: 2.2/5	PACIC: 2.5/5
Gender: female	Gender: female
Age: 49 years	Age: 61 years
Disease duration: 37 years	Disease duration: 2 years
Disease subset: dcSSc	Disease subset: lcSSc
Number of comorbidities: 3	Number of comorbidities: 1
**Low quality of life (i.e., high SScQoL score)**	**High quality of life (i.e., low SScQoL score)**
**High chronic care (i.e., high PACIC score)**	**High chronic care (i.e., high PACIC score)**
*Patient 3*	*Patient 4*
SScQoL: 27/29	SScQoL: 2/29
PACIC: 3.8/5	PACIC: 5.0/5
Gender: male	Gender: female
Age: 73 years	Age: 44 years
Disease duration: 13 years	Disease duration: 7 years
Disease subset: dcSSc	Disease subset: unknown
Number of comorbidities: 6	Number of comorbidities: 0

dcSSc, diffuse cutaneous systemic sclerosis; lcSSc, limited cutaneous systemic sclerosis; PACIC, Patient Assessment of Chronic Illness Care; SScQoL, Systemic Sclerosis Quality of Life

#### Data collection

Semi-structured individual interviews were conducted in German, French or English and were conducted (30–90 min in duration) either on-site or via telephone (due to COVID-19 pandemic restrictions) [38]. Open-ended interview questions (e.g., How do you experience your care? What would the best possible care look like for you?) were drawn from the CCM and patient’s narratives [20, 38]. The complete interview guide is published in the *MANOSS* study protocol [38]. Interviews were recorded and transcribed verbatim.

Focus group participants (n = 4) were engaged using an interview guide with open-ended prompts to discuss our quantitative study results (i.e., What is important/surprising? What fits your experience? What contradicts your own experience? What are important aspects that should be taken into account when improving chronic care for patients?). Subsequently, primary care needs and problem areas for care were discussed from a patient perspective. Due to the COVID-19 pandemic, the focus group was conducted using an online video conferencing (Zoom) and recorded with participant consent.

#### Qualitative data analysis

We used a reflexive thematic analysis approach described by Braun and Clarke [53, 54]. Briefly, investigators started analysis of interview transcripts by (1) familiarizing themselves with the data (i.e., reading and discussing first impression, main issues from patient perspective), (2) coding the data and developing first patterns of shared meaning across all interviews (i.e., inductive, but not theory free) and (3) constructing patterns/themes to explain PACIC dimensions. Finally, themes were refined and named based on original data (i.e., quotes, codes).

### Mixed methods data integration

The quantitative data informed the structure of the qualitative study. Subsequently, the qualitative data were used to explain the quantitative findings. Importantly, the mixed methods approach provides deeper insight for model development than either method in isolation [39]. We present our quantitative results first, followed by a joint display including key quantitative findings and qualitative in-depth themes for each PACIC dimension and data integration at the level of discussion.

## Results

### Participants’ characteristics

In total, 101 patients (median age = 60 yrs., IQR: 50–68) with a median disease duration of 8 years (IQR: 5–15) completed the survey (Table 2). Approximately half of patients (52/101, 51.5%) reported having more than two comorbidities. In total, 8 patients (interview: n = 4, focus group: n = 4) participated in the qualitative study. All four focus group participants were active members of a patient organization and three had a medical and/or scientific background, one participant had a rare rheumatic disease other than SSc since childhood.

**Table 2:** Patient characteristics of quantitative and qualitative study phases

Patients	Quantitative survey (n = 101)	Individual interviews (n = 4)	Focus group (n = 4)
Sex [n (%)]			
Female	77 (76.2%)	3 (75%)	4 (100%)
Male	20 (19.8%)	1 (25%)	
Not reported	4 (4%)		
Age (years) [median (IQR)]	60 (50–68)	55 (48–64)	57 (51–63)
Disease subset, self-reported [n (%)]			
lcSSc	31 (30.7%)	1 (25%)	1 (25%)
dcSSc	36 (35.6%)	2 (50%)	2 (50%)
Other rare rheumatic disease	3 (3.0%)		1 (25%)
Don’t’ know	25 (24.8%)	1 (25%)	
Disease duration (years) [median (IQR)]	8 (5–15)	10 (6–19)	32 (17–40)
Comorbidities, self-reported [median (IQR)]	3 (1–4)	2 (1–4)	
Questionnaire [n (%)]			
Online survey	43 (42.6%)		
Paper survey	58 (57.4%)		
Country/Region of origin [n (%)]			
Switzerland (German region)	79 (78.2%)		
Switzerland (French region)	22 (21.8%)		
Marital status [n (%)]			
Single	13 (12.9%)		
Married/cohabiting	68 (67.3%)		
Divorced, separated or widowed	16 (15.9%)		
Not reported	4 (4%)		
Highest educational degree [n (%)]			
Tertiary level	37 (36.7%)		
Upper secondary	48 (47.5%)		
Compulsory	13 (12.9%)		
No completed education	1 (1%)		
Not reported	2 (2%)		
Employment [n (%)]			
Employed	50 (49.5%)		
Full time (80–100%)	22 (21.8%)		
Part time (< 80%)	28 (27.9%)		
Reasons for non-employment [n (%)]			
Retired	30 (29.7%)		
On disability or sick leave	10 (9.9%)		
In training/student	7 (7%)		
Looking for work	4 (4%)		

dcSSc, diffuse cutaneous systemic sclerosis; IQR, interquartile range; lcSSc, limited cutaneous systemic sclerosis; SScQoL, Systemic Sclerosis Quality of Life

### Patient Assessment of Chronic Illness Care (PACIC) and its associations with patient characteristics, comorbidities and quality of life (HRQoL)

The distribution of all PACIC-15 scales in the overall, the German and the French-speaking *MANOSS* sample (n = 101) is presented in Table 3. Single item values are presented in Table 4 (i.e., joint display of quantitative and qualitative findings). The mean overall PACIC-15 score was relatively low (${\overline{x}}$ = 3.0, 95% CI 2.8–3.2, n = 100) indicating that care was ‘never’ to ‘generally not’ perceived as aligned with the CCM. Lowest PACIC-15 mean subscale scores related to ‘goal setting/tailoring’ (${\overline{x}}$ = 2.5, 95% CI 2.2–2.7, n = 99), followed by ‘problem solving/contextual counselling’ (${\overline{x}}$ = 2.9, 95% CI 2.7–3.2, n = 99). The single PACIC-15 items with the lowest ratings were: ‘given a copy of my treatment plan’ (${\overline{x}}$ = 2.0, 95% CI1.7–2.3, n = 97) and ‘helped to plan ahead so I could take care of my condition(s) even in hard times’ (${\overline{x}}$ = 2.5, 95% CI 2.2–2.8, n = 98).

**Table 3:** Distribution of the 15-item PACIC scale

PACIC scales	PACIC mean scores (95% CI)		
	Overall (n = 101)	German sample (n = 79)	French sample (n = 22)
PACIC 15-item scale summary score (average of all 15 items)	3.0 (2.8–3.2)	3.1 (2.9–3.4)	2.7 (2.2–3.2)
*Subscale 1* Patient activation (average of items 1–3)	3.4 (3.1–3.6)	3.4 (3.2–3.7)	3.0 (2.4–3.6)
*Subscale 2* Delivery system design/Decision support (average of items 4–6)	3.2 (3.0–3.4)	3.3 (3.0–3.5)	3.2 (3.0–3.4)
*Subscale 3* Goal setting/Tailoring (average of items 7–9)	2.5 (2.2–2.7)	2.6 (2.3–2.9)	1.9 (1.4–2.4)
*Subscale 4* Problem solving/Contextual counselling (average of items 12–15)	2.9 (2.7–3.2)	3.0 (2.7–3.3)	2.7 (2.1–3.3)
*Subscale 5* Follow-up/Coordination (average of items 19–20)	3.3 (3.0–3.5)	3.3 (3.0–3.7)	2.9 (2.3–3.6)

CI, confidence interval; PACIC, Patient Assessment of Chronic Illness Care

**Table 4:** Joint display of key quantitative findings for each PACIC subscale and interrelated qualitative theme

Main quantitative results	Description of qualitative themes and quotes
**PACIC dimension: Delivery system design/Decision support** *Definition*: Actions that organize care and provide information to patients to enhance their understanding of care **PACIC dimension: Patient activation** *Definition*: Actions that solicit patient input and involvement in decision-making	
**Delivery system design/Decision support **(Item 4–6)	*Theme 1: Experiencing organized care with limited participation *Describes SSc patients’ experiences with care delivery and shared decisions*
Most patients were satisfied with overall organisation of care (Item 5: ${\overline{x}}$ = 3.9, 95% CI 3.7–4.1) Nevertheless, only 29% of patients (always/most of the time) received a written list of things they should do to improve their health (Item 4: ${\overline{x}}$ = 2.6, 95% CI 2.3–2.8) Only 37% were (always/most of the time) shown how their self-management strategies influenced their condition (Item 6: ${\overline{x}}$ = 3.1, 95% CI 2.8–3.3) Differences in reported PACIC levels were found according to patient comorbidities. Patients with lung problems reported the highest mean PACIC levels (${\overline{x}}$ = 3.2, CI: 2.9–3.5), while lower levels were found in those with more than 2 comorbidities (${\overline{x}}$ = 2.8, CI: 2.5–3.0)	Participants appreciated regular medical check-ups (approx. every 1–2 years), but feared negative results. Those with an early/mild form of SSc sometimes doubted the necessity of such expensive examinations *«The examinations were really stressful. The organization was super, but I thought, hopefully they won’t find anything. The more examinations there are, the sicker you feel. I thought, I just have to hang in there. But I asked myself later if that was really necessary. If they don’t find anything, fine—but it still costs a lot. It’s contradictory, or..?!» (Patient 4, interview)* Participants reported limited own participation during consultations/check-ups. They all reported to be able to ask questions, but in their perception, it was the healthcare team that made the decisions *«Later, the (health care professionals) talked among themselves and reached an agreement, then I was asked to join them and was informed. That’s good in principle, then I can ask questions or learn what’s going to be done next.» (Patient 1, interview)* Participants experienced that their own self-management strategies were not specifically valued or integrated by healthcare professionals (HPs). Some reported being afraid to inform physicians about strategies such as complementary therapies or felt not taken seriously if they did *«I noticed myself that it’s hard to contribute during the examination. For about 10 years I didn’t dare to say I take Vitamin D. And now I also take B-Vitamins.» (Patient focus group)* One patient described her experiences with one-to-one peer support as very supportive in the decision-making process for a possible lung transplantation *«Lung transplantation was being considered for me. I could talk to two people who had been through that. With one person in a one-to-one telephone call and I met the other personally. I got a lot out of it because it was possible to talk about personal problems and ask questions. You don’t dare do that in a group.» (Patient 2, interview)*
**Patient activation** (item 1–3)	*Theme 2: Dealing with the illness in tailored measure *Describes patients being overwhelmed and protecting themselves from constant confrontation with their disease*
*Quantitative results*: 27–31% of patients with SSc were never/generally not asked for their ideas when a treatment plan was made nor given choices about treatment to think about (Item 1: ${\overline{x}}$ = 3.6, 95% CI 3.3–3.8, Item 2: ${\overline{x}}$ = 3.2, 95% CI 2.9–3.4)	Participants reported being overwhelmed with the disease information they received just after diagnosis, especially when it came in a written form without much explanation and the possibility to ask questions *«I had questions (about the disease) and since I knew nothing about it, he (doctor) just gave me a brochure. I knew I have a limited system sclerosis, but [the brochure] described really everything horrible and that scared me even more. It was terrible. I felt awful for a while and didn’t read anything more. I had first to get over the shock.» (Patient 2, interview)*
Only half of patients were (always/most of the time) asked to talk about their problems with medicines or their effects (Item 3: ${\overline{x}}$ = 3.4, 95% CI 3.1–3.6)	Participants who did not experience their symptoms as part of a severe disease, especially if the symptoms were only mild, did not want to deal with examinations and possible disease consequences, which have not yet occurred. Nevertheless, several emphasized the need for a step-wise learning process oriented toward progression of their own illness *«I try to live as normally as possible. That’s why I don’t do some things that make me feel sick (for example, examinations) even if that overtaxes me sometimes. I like it when I can solve something myself or can call in if something is wrong.» (Patient 1, interview)*
	Participants in a later disease stage described how they protect themselves from a constant confrontation with the disease by a reduction of medical consultations and examinations or by limiting them to a certain period of time. Those with extensive expertise reflected that for optimal patient activation, health care professionals have to tailor information and provide support according to an individual patient’s disease stage and readiness *«At the beginning, you really need support from other people. Later there’s a phase where you can manage the situation yourself. But later, you reach another point where you just can’t cope, because this happens or that turns up. I am now at a point where I have lost the orientation. I don’t know any more what I should do, or which doctor I should see. Then you need support again. What you need differs and it’s really hard for the health care professionals. The only solution is talk to one another and find out what is difficult for the person and what can be done about it. Just general strategies don’t work.» (Patient focus group)*
**PACIC dimension: Goal setting/Tailoring** *Definition*: Acquiring information for and setting of specific, collaborative goals **PACIC dimension: Problem-solving/Contextual Counselling** *Definition*: Considering potential barriers and the patient’s social and cultural environment in making treatment plans	
**Goal setting/Tailoring** (Item 7–11)	*Theme 3: Not knowing which strategies are effective or harmful *Describes the lack of guidance for independent self-management by patients*
*Quantitative results:* 73% of patients never/generally not received a copy of their treatment plan (Item 9: ${\overline{x}}$ = 2.0, 95% CI 1.7–2.3) 60% were never/generally not encouraged to go to a patient support group or class (Item 10: ${\overline{x}}$ = 2.3, 95% CI 2.0–2.6) Almost half of patients were never/generally not asked to talk about their self-management goals (43%) or helped to set specific goals to improve their eating or exercise (47%) (Item 7: ${\overline{x}}$ = 2.8, 95% CI 2.5–3.0, Item 8: ${\overline{x}}$ = 2.6, 95% CI 2.4–2.9) 22% were never/generally not asked questions about their health habits (e.g., risk factors such as smoking) (Item 11: ${\overline{x}}$ = 3.6, 95% CI 3.3–3.9) **Problem-solving/Contextual counselling **(Item 12–15) *Quantitative results:* 57% of patients were (always/most of the time) sure that their doctor or nurse thought about their values and traditions when recommending a treatment (Item 12: ${\overline{x}}$ = 3.5, 95% CI 3.3–3.8) 55% were never/generally not helped to plan ahead to take care of their condition(s) even in hard times (Item 14: ${\overline{x}}$ = 2.5, 95% CI 2.2–2.8), nor were they helped to make a treatment plan that for their daily life (48%, Item 13: ${\overline{x}}$ = 2.7, 95% CI 2.4–3.0) 15% (n = 15) reported having a depression in the last year	Participants described that they lacked guidance and exchange with health care professionals on self-management strategies to maintain their health and well-being, not only during and after diagnosis, but also later when they were already considered ‘experienced’ patients *«I always had the feeling that the answer (from the doctor about what one can do oneself) was that the course of the disease is very individual and for that reason, there is no general answer. That doesn’t really help and I said to myself: OK, I’ll just leave it.» (Patient 2, interview)* Participants explained that even when self-management strategies were recommended by professionals, as for example to improve physical activity, they often felt lost with respect to planning and evaluating these activities. Even participants spending time searching for appropriate information reported difficulties in adapting the information they found *«They never told me what I really should do with the crosstrainer or the bicycle in reference to me specifically, how long, at what level, at what watt setting. I’m sorry about that, because I really don’t know whether I do too much or too little. It would also help as motivation to training. I don’t even know if it would be noticed, if I didn’t go there.» (Patient 1, interview)* Some participants questioned the strong focus on medical outcomes, such as lab or examination results, to evaluate their health and well-being. In particular experienced patients emphasized the importance of patient- experienced outcomes as a focus of care, to prevent anxiety and insufficient self-management in patients.*«The question is also: "Who defines the outcomes?" When the doctor looks at the values and says: "Super! It’s stayed the same, the blood value is good! That’s good, I am satisfied." But the patient feels worse. Yeah, what happens then?» (Patient focus group)*
	*Theme 4: Feeling left alone with disease and psychosocial consequences *Describes the difficulties of dealing with disease consequences that are rarely addressed by HPs*
	Participants reflected on their difficulties to address negative emotions and social problems with health professionals. They reported feelings of shame or just did not expect support from professionals. Especially patients in the early stages of the disease described how they isolated and tried to cope with negative emotions by themselves *«You isolate yourself in shock and in fear of what can happen. And then there is self-stigmatization. You think you’re alone in the world with this disease. And the professionals can’t help you, although they know everything.» (Patient focus group)* Participants revealed that psychosocial consequences such as depressive symptoms or financial problems were not systematically addressed by professionals. Several patients reported that they suffered from problems of feeling down, sleeplessness or financial worries over longer time periods and had not been asked about these by professionals *«What was my experience? Well, definitely not thorough, that someone (professional) said, so let’s sit down and talk about you and your situation, what effects this might have (..) In fact, my husband was the one who suffered most, who had to put up with everything I couldn’t explain. This despair: Now I am really sick and there is no cure and that’s terrible. And the sleeplessness and all these things, he had to put up with them. It certainly wasn’t an easy time for him.» (Patient 2, interview)* Participants emphasised the importance of having at least one trustworthy person to talk to about their daily concerns related to their disease. Some did not want to burden their relatives with these worries or did not feel understood by them and sought professional help themselves by consulting a psychologist or psychotherapist *«I can’t really talk about this at home. They don’t understand. That’s why I think psychological help is important, to get this off my chest. It’s not about finding new strategies to do things better, it’s about the everyday burden, everyday worries.» (Patient 1, interview)* Participants did not perceive self-help groups as a source of support for problem solving. Whereas some felt they were still 'too healthy' to join such a group, others experienced participation as additional emotional strain on them. For example, because of moaning by others. Given the variety of disease representations and experiences, focus group participants discussed the limitations of traditional self-help groups for people with SSc *«It doesn’t help. I come out of there and say, how can one ever be so taken by yourself, by the disease, that that’s the main topic in life. It is often very extreme, when you always hear the same thing at every meeting, I just have to say, that’s too much for me.» (Patient 1, interview)*
**PACIC dimension: Follow-up/Coordination** *Definition*: Arranging care that extends and reinforces office-based treatment, and making proactive contact with patients to assess progress and coordinate care	
**Follow-up/Coordination** (Item 16–20) *Quantitative results:* 69% were never/generally not encouraged to attend programs in the community (Item 17: ${\overline{x}}$ = 1.9, 95% CI 1.7–2.1) 64% were never/generally not contacted after a visit to see how things were going (Item 16: ${\overline{x}}$ = 2.1, 95% CI 1.8–2.4) 55% were never/generally not referred to a dietitian, specialist nurse/health professional (Item 18: ${\overline{x}}$ = 2.5, 95% CI 2.2–2.8) 42% were never/generally not asked how their visits with other doctors were going (Item 20: ${\overline{x}}$ = 3.0, 95% CI 2.6–3.3) 24% were never/generally not told how their visits with doctors like heart or vascular specialist helped their treatment (Item 19: ${\overline{x}}$ = 3.6, 95% CI 3.3–3.8)	**Theme 5: Taking over complex coordination of care *Describes how difficult it is for patients to obtain a problem/therapy synthesis and to coordinate their own care*
	Participants emphasized that living with a rare disease such as SSc requires consultations with many specialized medical providers. Timewise this required coordinating work by themselves. Overall, they perceived these services as highly competent with some variety in professional’s ability to summarize results in plain understandable language. This was important for them, to make sense out of the examination results in a specific organ-oriented field *«I had to take a lung function test every year and the information there was very good. I also thought highly of that doctor. He spent about half an hour with me after the test and explained what it means and what I have to expect, or maybe expect, in the future.» (Patient 2, interview)* Participants reported that the communication of an overall synthesis (i.e., of the many specialists’ examination results and decisions) to gain an overview of individual disease manifestations and outcomes was lacking. They highlighted their need and attempts for a coordinated decision-making, by bringing information from other services into discussions. However, all emphasized that these coordination tasks cannot be conducted equally well by all patients at all time points as it needs expertise, self-empowerment and depends on disease-complexity *«That’s how it is in the hospital, I understand that, they have a lot to do, but then someone comes about blood vessels, then someone about the lungs, and then there’s someone about the heart, totally different things. And there is no summery at the end. I am no professional and I can’t put the puzzle together from these pieces.» (Patient 2, interview)* Remarkably, some highly experienced patients reported that they took over the coordination of the involved professionals (e.g., organ specialists, other HPs) and made them aware of their multiple problems to be considered in the overall decision making. This gave them a sense of control *«It was important to me to realize that the disease affects the whole system, everything, physical, emotional, family, material. And our health system is fragmented and organ-related. There is no Care Management or Case Management, at least not in Switzerland. And I had to learn on my own to do that, to see how I can deal with the specialists. These specialists who pounce on one organ system and know a whole lot about that, but don’t see the connection or only want maximum therapy for one part. Who pulls this all together if I don’t? After all, we can’t change the system so quickly. We can only emancipate ourselves.» (Patient focus group)* Additionally, some participants highlighted the problem of short-coming economic incentives in our fragmented healthcare system, which prevents care coordination and long-term savings *«I need 3* × *50 mg per day. And then I found out that there is also Sildenafil from Sandoz, 3* × *50 and I figured out what that costs for a year. The Revatio, 20 mg, or Sandoz 50 mg. Sandoz 50 mg costs almost 10,000 Franken less per year. I blame the whole system a little. There are things that could be optimized using these doses or brands. I would think looking at such things would be part of the medication check the pharmacies make.» (Patient 3, interview)*
	**Theme 6: Relying on an accessible and trustworthy team *describes patients’ initiatives to find reliable professionals and peers to support them on an ongoing basis*
	Participants highlighted that the ‘rigid’ annual monitoring appointments have the advantage that examinations with a range of relevant specialists are bundled and coordinated within one centre of expertise. This enables easy and reliable access to a defined range of providers once a year. However, individual adaptations to this plan (e.g., meetings with additional professionals for specific problems) were only possible to a limited extent *«I like having an assessment where there is a location once a year. I don’t live directly near the hospital and it’s really great for me if I don’t always have to make the trip. But what isn’t so good, you can’t always introduce additional things. Even if you said you wanted to when the date was set.» (Patient 1, interview)* Building their own trustworthy team of professionals was a major point raised by participants to improve care. Having confidence in their expertise and being cared for continuously over a long period of time was crucial for them. They even paid for consultations and therapies not reimbursed by the health insurance out of their own pockets to collaborate with professionals of their preference and receive the therapies they needed *«With respect to Physio, I organized something special for myself. I think the person is very competent, so the relationship is very good. It is not only Physio, but sometimes a bit psychological as well. We’ve known each other for quite a while. But that’s it, when you know each other for some time—now with that one person it’s not the problem, with the other it is the problem: it becomes routine. And then the same thing is done, whether you need it or not.» (Patient 1, interview)* Participants in a later disease stage described how they had learned to educate professionals who were not yet familiar with the disease and its consequences. In this context, they highlighted the importance of self-management support to reach expertise. Trustworthiness of an individual provider was finally also judged on their ability to collaborate with them *«I see it as a tipping point, a kind of transition where you are quasi the expert and you get asked and you’re "empowered" in the sense: I now look after myself. And if I have to go somewhere else, like to a new dentist or a new gynaecologist, then I tell the doctor that I have this disease and that I have dry mouth, for example. The doctor maybe never heard of sclerodermatitis and I can explain it…» (Patient focus group)*

*Qualitative theme accounting for two PACIC dimensions

**Qualitative theme accounting for one PACIC dimension

***PACIC: 5-point scale (1 = ‘never’ to 5 = ‘always’)

Overall, patient characteristics were not associated with individual (mean) PACIC-15 scores. Considering comorbidities, only self-report of lung problems showed a significant difference in mean PACIC-15 scores (Table 5). However, patients ≤ 65 years (${\overline{x}}$ = 3.1 vs. 2.7; SMD = 0.41, n = 95) and males (${\overline{x}}$ = 3.3 vs. 3.0; SMD = 0.33, n = 97) reported higher mean PACIC scores. Patients trended towards lower mean PACIC scores early in the disease trajectory (i.e., within two years of diagnosis) (${\overline{x}}$ = 2.9 vs. 3.1; SMD = 0.15, n = 95) and in subgroup with diffuse cutaneous systemic sclerosis (dcSSc) (${\overline{x}}$ = 2.9 vs. 3.2; SMD = 0.26, n = 67). Interestingly, patients with lung (${\overline{x}}$ = 3.4 vs. 2.8; SMD = 0.61, n = 100) and gastrointestinal (GI) problems (${\overline{x}}$ = 3.2 vs. 2.9; SMD = 0.29, n = 100) reported higher PACIC levels than those without pulmonary/GI comorbidities. Patients with musculoskeletal complaints reported lower PACIC scores (back pain: ${\overline{x}}$ = 2.9 vs. 3.2; SMD = 0.31, n = 97; osteoarthritis: ${\overline{x}}$ = 2.8 vs. 3.1; SMD = 0.30, n = 98). Further, patients with more than two self-reported comorbidities reported lower PACIC levels (${\overline{x}}$ = 2.8 vs. 3.0; SMD = 0.28, n = 100).

**Table 5:** Univariate analyses of patient characteristics and comorbidities in relation to the mean PACIC-15 score (n = 101)

	Characteristics	Mean PACIC score (95% CI)	*P* value	SMD
Sex	Male (n = 20)	3.3 (2.8–3.9)	0.172	0.326
	Female (n = 77)	3.0 (2.7–3.2)		
Age	≤ 65 years (n = 65)	3.1 (2.9–3.4)	0.063	0.408
	> 65 years (n = 30)	2.7 (2.3–3.1)		
Education	Compulsory/no education (n = 14)	3.2 (2.4–3.9)	0.617	0.133
	Secondary/tertiary (n = 85)	3.0 (2.8–3.2)		
Marital status	Single, divorced, or widowed (n = 29)	3.2 (2.8–3.6)	0.24	0.259
	Married/cohabiting (n = 68)	2.9 (2.7–3.2)		
Disease subset*	dcSSc (n = 36)	1.9 (2.6–3.2)	0.284	0.263
	lcSSc (n = 31)	3.2 (2.8–3.6)		
Disease duration*	≤ 2 years (n = 12)	2.9 (2.4–3.5)	0.637	0.153
	> 2 years (n = 83)	3.1 (2.9–3.3)		
Comorbidities*	≤ 2 comorbidities (n = 48)	3.2 (2.9–3.5)	0.176	0.273
	> 2 comorbidities (n = 52)	2.9 (2.6–3.2)		
Depression*	Yes (n = 15)	3.0 (2.4–3.5)	0.708	0.107
	No (n = 84)	3.1 (2.8–3.3)		
GI-problems*	Yes (n = 60)	3.2 (2.9–3.4)	0.149	0.294
	No (n = 40)	2.9 (2.5–3.2)		
Lung problems*	Yes (n = 38)	3.4 (3.1–3.7)	**0.004**	0.607
	No (n = 62)	2.8 (2.6–3.1)		
Heart problems*	Yes (n = 27)	3.0 (2.6–3.4)	0.816	0.053
	No (n = 72)	3.0 (2.8–3.3)		
Backpain*	Yes (n = 41)	2.9 (2.6–3.1)	0.132	0.315
	No (n = 56)	3.2 (2.9–3.4)		
Osteoarthritis*	Yes (n = 41)	2.8 (2.6–3.1)	0.151	0.299
	No (n = 57)	3.1 (2.9–3.4)		

CI, confidence interval; dcSSc, diffuse cutaneous systemic sclerosis; GI-problems, gastrointestinal problems; lcSSc, limited cutaneous systemic sclerosis; PACIC, Patient Assessment of Chronic Illness Care; SD, standard deviation; SMD, standardized mean difference (SMD ≥ 0.2, ≥ 0.5 and ≥ 0.8 depict small, medium and large differences between groups respectively); numbers in bold = p-values < 0.05

*Self-reported

No significant correlations (pearson’s *r*) were identified between the mean PACIC-15 and SScQoL scores (neither total score nor sub-dimensions) (see Additional file 2).

### Association of HRQoL and patient characteristics/comorbidities

The overall mean SScQoL score was 18.3 (95% CI 16.7–19.9). Patients from German-speaking Switzerland tended to have better SScQoL outcomes (${\overline{x}}$ = 17.4 vs. 21.4; SMD = 0.56), particularly in ‘emotional’ (${\overline{x}}$ = 7.5 vs. 9.9; SMD = 0.71) and ‘sleep’ (${\overline{x}}$ = 1.2 vs. 1.7; SMD = 0.61) dimensions. Younger patients (≤ 65 years) tended to report poorer HRQoL (${\overline{x}}$ = 19.0 vs. 16.6; SMD = 0.30) (Table 6).

**Table 6:** Distribution of the 29-item SScQoL scales

SScQoL scales	SScQoL mean scores (95% CI)		
	Overall (n = 101)	German sample (n = 79)	French sample (n = 22)
SScQoL 29-item scale summary score (range 0–29)	18.3 (16.7–19.9)	17.4 (15.5–19.3)	21.4 (19.0–23.8)
*Subscale 1* Function (range 0–6)	4.1 (3.8–4.4)	4.0 (3.6–4.4)	4.5 (4.0–5.1)
*Subscale 2* Emotional (range 0–13)	8.0 (7.3–8.8)	7.5 (6.6–8.4)	9.9 (8.8–10.9)
*Subscale 3* Sleep (range 0–2)	1.3 (1.1–1.5)	1.2 (1.0–1.4)	1.7 (1.4–2.0)
*Subscale 4* Social (range 0–6)	3.5 (3.1–3.9)	3.5 (3.0–4.0)	3.7 (2.9–4.6)
*Subscale 5* Pain (range 0–2)	1.3 (1.1–1.5)	1.2 (1.0–1.4)	1.6 (1.3–1.9)

CI, confidence interval; SScQoL, Systemic Sclerosis Quality of Life

SScQoL answer options are dichotomised for analysis: ‘Always’, ‘Usually’, ‘Sometimes’ = 1; ‘Never’ = 0; Higher scores indicate a greater impact of the disease, i.e., decrease of health-related quality of life (HRQoL)

Notably, HRQoL was strongly associated with self-reported comorbidities but no other patient characteristics (Table 7). Neither sex, marital status nor disease subset/duration were associated with SScQoL mean score. Patients ≤ 65 years old (${\overline{x}}$ = 19.0 vs. 16.6; SMD = 0.30, n = 95) and with compulsory or no education (${\overline{x}}$ = 20.3 vs. 18.0; SMD = 0.30, n = 99) tended to exhibit lower HRQol (i.e., higher SScQoL scores). The number of patient self-reported comorbidities had a deleterious influence on SScQoL. Patients reporting more than two comorbidities (51.5%, n = 52) had lower HRQoL—as evidenced by significantly higher SScQoL score (${\overline{x}}$ = 22.3 vs. 14.3; SMD = 1.15, n = 100). Similar findings were observed in individuals reporting depression (${\overline{x}}$ = 24.3 vs. 17.4; SMD = 1.10, n = 99), gastrointestinal problems (${\overline{x}}$ = 21.2 vs. 14.3; SMD = 0.94, n = 100) and osteoarthritis (${\overline{x}}$ = 21.3 vs. 16.4; SMD = 0.64, n = 98).

**Table 7:** Univariate analyses of patient characteristics/comorbidities in relation to mean SScQoL score (n = 101)

	Characteristics	Mean SScQoL score (95% CI)	*P* value	SMD
Sex	Male (n = 20)	16.8 (12.7–21.0)	0.372	0.216
	Female (n = 77)	18.6 (16.9–20.4)		
Age	≤ 65 years (n = 65)	19.0 (17.1–21.0)	0.169	0.304
	> 65 years (n = 30)	16.6 (13.6–19.6)		
Education	Compulsory/no education (n = 14)	20.3 (16.5–24.0)	0.328	0.306
	Secondary/tertiary (n = 85)	18.0 (16.3–19.8)		
Marital status	Single, divorced, or widowed (n = 29)	17.3 (14.1–20.5)	0.374	0.194
	Married/cohabiting (n = 68)	18.9 (17.1–20.8)		
Disease subset*	dcSSc (n = 36)	20.9 (18.6–23.2)	0.232	0.294
	lcSSc (n = 31)	18.7 (15.7–21.7)		
Disease duration*	≤ 2 years (n = 12)	18.6 (13.0–24.2)	0.858	0.052
	> 2 years (n = 83)	19.0 (17.4–20.7)		
Comorbidities*	0–2 comorbidities (n = 48)	14.3 (12.1–16.6)	** < 0.001**	1.147
	> 2 comorbidities (n = 52)	22.3 (20.6–24.0)		
Depression*	Yes (n = 15)	24.3 (22.3–26.4)	**0.002**	1.097
	No (n = 84)	17.4 (15.6–19.2)		
GI-problems*	Yes (n = 60)	21.2 (19.6–22.9)	** < 0.001**	0.937
	No (n = 40)	14.3 (11.6–17.0)		
Lung problems*	Yes (n = 38)	19.6 (17.2–22.0)	0.262	0.236
	No (n = 62)	17.8 (15.6–19.9)		
Heart problems*	Yes (n = 27)	20.0 (17.2–22.9)	0.204	0.296
	No (n = 72)	17.8 (15.8–19.7)		
Backpain*	Yes (n = 41)	20.7 (18.2–23.2)	**0.018**	0.496
	No (n = 56)	16.8 (14.7–18.9)		
Osteoarthritis*	Yes (n = 41)	21.3 (19.0–23.6)	**0.002**	0.643
	No (n = 57)	16.4 (14.3–18.5)		

CI, confidence interval; dcSSc, diffuse cutaneous systemic sclerosis; GI-problems, gastrointestinal problems, lcSSc, limited cutaneous systemic sclerosis; SScQoL, Systemic Sclerosis Quality of Life; SD, standard deviation; SMD, standardized mean difference; numbers in bold = p-values < 0.05

*Self-reported

### Qualitative findings

The quantitative findings informed the structure of the qualitative data description—presented in a joint display (see Table 4). More concrete, patient experiences with the current chronic care approach are described in six themes illustrated with patient quotes. Whereas always two qualitative themes are mapped to the PACIC dimensions: (1) ‘*experiencing organized care with limited participation*’ and (2) ‘*dealing with the illness in tailored measure*’ (belonging to ‘patient activation’ and ‘delivery system design/decision support’); (3) ‘*not knowing which strategies are effective or harmful*’ and (4) ‘*feeling left alone with disease and psychosocial consequences*’ (belonging to ‘goal setting/tailoring’ and ‘problem solving/contextual counselling’); (5) ‘*taking over complex coordination of care*’ and (6) ‘*relying on an accessible and trustworthy team*’ (belonging to ‘follow-up/coordination’). In respect to Table 4, the reader is advised to start with the dimension definition, then the overview of the quantitative results followed by the qualitative results to better understand the patient experience.

## Discussion

In this investigation of SSc care, we found relatively low PACIC values overall. Patients identified the greatest deficits in the areas of ‘*goal setting/tailoring*’ and ‘*problem solving/contextual counselling*’*.* These observations are further supported by the qualitative findings that revealed significant need for SSc self-management support and care coordination, both key elements of CCM.

The low PACIC mean overall score of 3.0/5.0 (95% CI 2.8–3.2) in our study is comparable to findings in patients with common chronic diseases [26, 30, 36]. However, direct comparison of PACIC scores should be done with caution as slightly different version have been used across studies. A 2018 meta-analysis of 34 studies from 13 countries (> 25,000 patients with diabetes) [36] identified a pooled score of 3.0 (95% CI 2.8–3.2). Interestingly, a survey conducted by EURORDIS (a European alliance of 970 rare disease patient organisations from 74 countries) used the abbreviated 11-item PACIC [55] and found patients report a better chronic care experience (${\overline{x}}$ = 3.4 vs. 2.6) when treated in centres belonging to a European Reference Network (ERN)—highlighting the critical role for access to expert care for rare diseases.

Notably, we did not find an association between PACIC scores and HRQoL. However, mean SScQoL scores were significantly associated with a number of self-reported comorbidities (depression, gastrointestinal problems and osteoarthritis). Such findings are in line with studies of common chronic conditions, in which PACIC scores were marginally correlated with HRQoL (r = 0.15 and 0.23) [26, 28, 56]. Our observation is explained by qualitative investigation that revealed a number of factors influencing patient ratings of care (i.e., gratitude, faith, loyalty, luck, equity, engagement with the system) [57]. Interestingly, patients with lung problems reported higher PACIC levels than those without pulmonary complications. It is plausible that patients’ evaluation of care may depend on their perceived level of influence and engagement with the healthcare system—rather than HRQoL per se.

Nevertheless, PACIC dimensions can inform development or improvement of integrated models of SSc care [27]. In the present study, PACIC scores indicate shortcomings in ‘*goal setting/tailoring*’ and ‘*problem solving/contextual counselling*’. The patient-identified gaps in care pose significant barriers to effective self-management. Indeed, the described qualitative themes ‘*not knowing which strategies are effective or harmful*’ and ‘*feeling left alone with disease and psychosocial consequences*’ highlight the quantitative findings*.* Our observations are similar to studies in common chronic conditions that identified the same PACIC dimensions had the lowest mean values [26, 28]. Similarly, prior qualitative work in SSc, found that patients often lack guidance and effective strategies for independent self-management—particularly in relation to disease and psychosocial consequences [58–60]. Indeed, a systematic review of 26 qualitative studies in SSc identified that patients often feel ‘*alone and misunderstood*’ (i.e., fearful avoidance of fellow patients, invisible suffering) despite having the opportunity to meet other patients in support groups [60]. Comparisons at the item level reveal similarities with European patients with other rare diseases [55]. Swiss SSc patients in the present study were—similar to rare disease patients in Europe—rarely helped to plan ahead for self-management in challenging times (${\overline{x}}$ = 2.5 for both groups) or connected with disease-specific patient support groups (${\overline{x}}$ = 2.3 and 2.1 respectively) [55]. Importantly, patients in our study noted limitations of traditional peer support groups. The present findings underscore and expand on previously identified gaps in care for patients with SSc and emphasize the importance of eliciting patient-defined goals/outcomes, developing self-management programmes and re-envisioning traditional on-site peer support groups [16, 59–62]. When implementing integrated care, patients and professionals should agree on a joint treatment plan including individualized goals targeting the primary SSc manifestations and consequences. Importantly, patients need to understand the essential elements for their individual disease self-management and require tailored education across the specialities involved in care. Therefore, it is important to foster provider skills and implement programs supporting psychological and self-management support to enable patients to self-manage their condition on a day-to-day basis [55].

Like Desmedt et al. [26] and Stuber et al. [35], we observed relatively high PACIC scores in the dimensions ‘*patient activation*’ and ‘*delivery system design/decision support*’—suggesting that patient with SSc generally feel involved in care decisions. Compared to European rare disease patients, Swiss SSc patients are more likely to ‘*receive treatment choices to think about*’ (${\overline{x}}$ = 3.2 vs. 2.8) and consider their care as ‘well organized’ (${\overline{x}}$ = 3.9 vs. 3.5) [55]. Congruently, interviews revealed that patients who had regular medical follow-up perceived their care as ‘*super organised*’ despite a persistent fear of receiving negative results. However, the qualitative theme ‘*experiencing organized care with limited participation*’—suggests that patients did not feel involved in medical consultations and that decisions were primarily provider-driven. Moreover, the theme ‘*dealing with the illness in tailored measure*’ describes the importance of protecting patients from feeling overwhelmed in confronting SSc—a finding that adds to prior qualitative SSc research [60]. Our qualitative inquiry reflects the importance of soliciting patient input and involving patients in decision-making as well as arranging care to extend and reinforce office-based consultations. Thus, improving healthcare provider competencies in shared decision-making is a key target for effectively implementing integrated SSc care [63, 64]. Stocker et al. [17] highlighted the need for patient decision aids to foster more patient-focused communication and support high quality decisions that are both informed and aligned with patient needs, values and preferences. Furthermore, our study revealed that patients may feel strained by too much, untimely or frightening information and may therefore refuse certain tests, examinations or interventions. To overcome such barriers, timely access to specialized care (e.g., virtual expert consultations, cross-border healthcare, knowledge assets produced by centres of expertise) warrant consideration [55].

With regard to the PACIC dimension ‘f*ollow-up/coordination*’, we identified major gaps in the complex care coordination of SSc (i.e., discontinuity and lack of follow-up). Among Swiss SSc patients in this study, patients were more likely to reported receiving feedback and explanations about specialist visits and examinations compared to European rare disease patients (${\overline{x}}$ = 3.6 vs. 2.5) [55]. However, qualitative interviews with experienced patients revealed that patients often assume responsibility for complex care coordination themselves. The theme ‘*taking over complex coordination of care*’ underscored the difficulty patients experience coordinating their own care. Similar to European rare disease patients, Swiss SSc patients rarely had contact with their healthcare provider after a visit, potentially explained by suboptimal provider reimbursement for outpatient services in the Swiss health system [65]. Moreover, patients may be receiving care in centres/practices that lack expertise in this rare disease [55]. Importantly, rare disease patients who were treated in centres belonging to a European Reference Network (ERN) reported higher satisfaction with regard to ‘being contacted after a visit’ (${\overline{x}}$ = 2.8 vs. 2.1). Congruently, our interview participants described ‘*relying on an accessible and trustworthy team*’ as a central theme relating to finding trusted, reliable professionals and peers for ongoing care and support. Several studies have revealed similar gaps in SSc care delivery (i.e., lack of structured multidisciplinary collaboration, inadequately organized follow-up, poor patient-provider relationships [17, 59, 66–68]. Despite the positive impact the chronic care model has demonstrated on disease outcomes, rare disease care models rarely test multi-component interventions (e.g., patient education, patient-held medical records, specialist nurse-led care) in providing coordinated, ongoing, complex care [32, 69] and infrequently incorporate community-based resources [70, 71]. In diabetes and cancer, chronic care implementation has long utilized specialized nurses and peers for support, case-management and counselling to improve patient-centredness, satisfaction with care and clinical outcomes [72–75]. Additionally, capacity building within health systems may be needed for a more flexible approach to planning consultations (e.g., self-referrals for lab tests and consultations) as well as co-management by patients and professionals (e.g., personal health records) to improve patient access, promote empowerment and reduce travel requirements [15, 76, 77].

In summary, comprehensive SSc care demands a systematic approach that addresses physical and mental health concerns as well as social consequences/inequities throughout the disease course. A collaborative approach between patients and providers is paramount with shared responsibility for decision-making and goal setting to arrive at a joint treatment plan. Additionally, tailored therapeutic education is an essential component of comprehensive, holistic SSc care. In regard to care delivery and follow-up/coordination important targets include improving provider skills (e.g., decision-making, self-management support) and novel modes of care (e.g., decision aids, virtual consultations, specialized nurses, peer-to-peer support, self-referrals, personal health records) may help create a more person-centered approach to SSc care.

Relative strengths of this study include the comprehensive assessment of patient experiences and needs for SSc chronic illness care using both quantitative and qualitative data from patients spanning a range of disease experience (i.e., newly diagnosed until long diseases duration). The study also has a number of limitations. First, the sample size is relatively limited, yet 101 patients included in the quantitative survey is a sizeable cohort for a rare disease [78]. Similarly, the qualitative sample used to contextualise the PACIC data was small. The purposeful selection of these participants ensured us to depict the variable disease trajectory of SSc. However, experience with a new diagnosis may be underrepresented because all participants in the qualitative part had two or more years of disease experience. In addition, the PACIC has not been formally validated for SSc. The PACIC has been used in rare disease populations [55]—yet it is unclear how well this generic instrument assesses the challenges specific to rare disease care (e.g. lack of treatment options and specialized healthcare professionals) and disease-specific patient needs. Prior research in common chronic diseases suggest that the single PACIC score is an appropriate measure of global chronic care—yet it is difficult to distinguish between the five PACIC dimensions [41, 79]. Unlike previous validation studies using confirmatory factor analysis, we applied Mokken Scale Analysis that relates to nonparametric Item Response Theory (IRT) models and is more appropriate for non-normally distributed data [42, 80]. Our validation revealed five items of the PACIC-20 dimensions not fitting our data. After excluding these problematic items, *H* coefficients were found to be strong for the global (0.52) and subscales (0.69, 0.70) suggesting a robust unidimensional scale (see Additional file 1). However, from a clinical perspective, excluding these items may be controversial—as considering scalability coefficients alone may yield an incomplete picture [42]. Indeed, patient care experiences with regard to peer support (e.g., item 10), follow-up (e.g., item 17) and referral to HPs (e.g., item 18) would be important for quality assessment of SSc and rare disease care in general.

## Conclusions

In summary, re-envisioning current SSc care practices and incorporating components of the Chronic Care Model (CCM) offer opportunities to improve chronic disease management of SSc patients in Switzerland. Our findings suggest that shared decision-making, goal-setting and tailored counselling are needed to better support patients to develop self-management skills. New models of care must focus on coordinating the complex care (including ongoing follow-up), and facilitating patients and professionals in sharing a leadership role to improve patient-centredness, satisfaction with care and clinical outcomes. Establishing more flexible approaches to scheduling consultations and fostering co-management by patients and professionals merits attention (e.g., specialized nurse-led case management and peer-to-peer counselling). Future research would be needed to receive a valid and reliable measure for the assessment of chronic illness care in rare diseases as SSc. Additional investigation may focus on comparing and contrasting centres providing care for people living with SSc and other rare (rheumatic) diseases to discern the key elements of chronic illness management for these populations.

## Supplementary Information

**Additional file 1**.** Table 1a**. Mokken scale analysis of global scale.** Table 1b**. Mokken scale analysis of subscales.** Table 2a**. Mokken scale analysis of global scale.** Table 2b**. Mokken scale analysis of subscales

**Additional file 2**. Correlation matrix (pearson’s r) of mean PACIC-15 and SScQoL scores.

## Data Availability

The datasets generated and/or analysed during this study are included in this published article or can be made available from the corresponding author on reasonable request.
