# Supplementary material for: Patient Assessment Chronic Illness Care (PACIC) and its associations with quality of life among Swiss patients with systemic sclerosis: a mixed methods study
Source: Orphanet J Rare Dis. 2023 Jan 9;18:7. doi: 10.1186/s13023-022-02604-2 (PMC9828378; doi:10.1186/s13023-022-02604-2)
Supplement: Supplementary file 1 — Additional file 1. Table 1a. Mokken scale analysis of global scale. Table 1b. Mokken scale analysis of subscales. Table 2a. Mokken scale analysis of global scale. Table 2b. Mokken scale analysis of subscales [file 13023_2022_2604_MOESM1_ESM.docx]

**Additional file 1**

***Validation of the PACIC questionnaire for SSc***

Because PACIC has not been used in the context of SSc, we used the Mokken model to test the construct validity of the PACIC scale and its subscales (49). Briefly, a scalability (*H* coefficient) value of ≥ 0.50 is considered ‘strong’, 0.40–0.49 = “moderate” and 0.30–0.39 = ‘weak’, and values < 0.30 are not considered unidimensional. Scalability for the global PACIC scale was “moderate” (0.46), with several ‘weak’ items (*H* coefficient below 0.4) (see Additional file 1, **Table 1a**). When items were grouped in respective subscales *H* coefficients were ‘strong’ in four subscales (‘patient activation’ [0.66], ‘delivery system design’ [0.60], ‘goal setting’ [0.50], ‘problem solving’ [0.67]), while ‘follow-up/coordination’ had ‘moderate’ scalability (0.42) (see Additional file 1, **Table 1b**). After excluding the problematic items of the ‘goal setting’ subscale (items 10 and 11) and ‘follow-up/coordination’ subscale (items 16, 17, 18), *H* coefficients were ‘strong’ for the subscales (0.69 and 0.70 respectively) and the global scale (0.52) suggesting a robust unidimensional scale (see Additional file 1, **Table 2a/2b**).

*Mokken scale analysis of the original 20-item Patient Assessment of Chronic Illness Care (PACIC)*

| **Table 1a.** Mokken scale analysis of global scale | | | |
| --- | --- | --- | --- |
|  | **Items** | **Scalability (*H*)** | **Standard error** |
| ***Global scale*** |  | **0.461** | **0.040** |
|  | Item 1 | 0.463 | 0.057 |
|  | Item 2 | 0.420 | 0.058 |
|  | Item 3 | 0.460 | 0.052 |
|  | Item 4 | 0.493 | 0.053 |
|  | Item 5 | 0.490 | 0.065 |
|  | Item 6 | 0.546 | 0.046 |
|  | Item 7 | 0.552 | 0.046 |
|  | Item 8 | 0.559 | 0.044 |
|  | Item 9 | 0.490 | 0.063 |
|  | Item 10 | 0.339 | 0.069 |
|  | Item 11 | 0.391 | 0.064 |
|  | Item 12 | 0.535 | 0.046 |
|  | Item 13 | 0.581 | 0.039 |
|  | Item 14 | 0.511 | 0.053 |
|  | Item 15 | 0.472 | 0.064 |
|  | Item 16 | 0.392 | 0.077 |
|  | Item 17 | 0.335 | 0.080 |
|  | Item 18 | 0.371 | 0.065 |
|  | Item 19 | 0.340 | 0.073 |
|  | Item 20 | 0.449 | 0.056 |
| **Table 1b.** Mokken scale analysis of subscales | | | |
|  | **Items** | **Scalability (*H*)** | **Standard error** |
| ***Patient activation*** |  | **0.664** | **0.059** |
|  | Item 1 | 0.720 | 0.050 |
|  | Item 2 | 0.677 | 0.060 |
|  | Item 3 | 0.595 | 0.075 |
| ***Delivery System Design/ Decision Support*** |  | **0.598** | **0.065** |
|  | Item 4 | 0.593 | 0.073 |
|  | Item 5 | 0.605 | 0.073 |
|  | Item 6 | 0.596 | 0.068 |
| ***Goal setting/ Tailoring*** |  | **0.502** | **0.061** |
|  | Item 7 | 0.545 | 0.064 |
|  | Item 8 | 0.596 | 0.054 |
|  | Item 9 | 0.524 | 0.071 |
|  | Item 10 | 0.418 | 0.078 |
|  | Item 11 | 0.423 | 0.079 |
| ***Problem solving/ Contextual counselling*** |  | **0.673** | **0.054** |
|  | Item 12 | 0.682 | 0.052 |
|  | Item 13 | 0.697 | 0.053 |
|  | Item 14 | 0.698 | 0.056 |
|  | Item 15 | 0.616 | 0.075 |
| ***Follow-up/ Coordination*** |  | **0.418** | **0.053** |
|  | Item 16 | 0.368 | 0.073 |
|  | Item 17 | 0.312 | 0.081 |
|  | Item 18 | 0.383 | 0.067 |
|  | Item 19 | 0.552 | 0.052 |
|  | Item 20 | 0.462 | 0.059 |

Table legend: scalability *H* ≥ 0.50 = strong, 0.49 to 0.40 = moderate, 0.39 to 0.30 = weak, while values of < 0.30 are not considered as unidimensional.

*Mokken scale analysis of the adapted 15-item Patient Assessment of Chronic Illness Care (PACIC)*

| **Table 2a.** Mokken scale analysis of global scale | | | |
| --- | --- | --- | --- |
|  | **Items** | **Scalability (*H*)** | **Standard error** |
| ***Global scale*** |  | **0.521** | **0.042** |
|  | Item 1 | 0.497 | 0.058 |
|  | Item 2 | 0.460 | 0.059 |
|  | Item 3 | 0.511 | 0.054 |
|  | Item 4 | 0.506 | 0.058 |
|  | Item 5 | 0.545 | 0.065 |
|  | Item 6 | 0.564 | 0.051 |
|  | Item 7 | 0.591 | 0.047 |
|  | Item 8 | 0.584 | 0.046 |
|  | Item 9 | 0.520 | 0.065 |
|  | Item 12 | 0.566 | 0.048 |
|  | Item 13 | 0.621 | 0.040 |
|  | Item 14 | 0.559 | 0.054 |
|  | Item 15 | 0.494 | 0.068 |
|  | Item 19 | 0.323 | 0.081 |
|  | Item 20 | 0.475 | 0.061 |
| **Table 2b.** Mokken scale analysis of subscales | | | |
|  | **Items** | **Scalability (*H*)** | **Standard error** |
| ***Patient activation*** |  | **0.664** | **0.059** |
|  | Item 1 | 0.720 | 0.050 |
|  | Item 2 | 0.677 | 0.060 |
|  | Item 3 | 0.595 | 0.075 |
| ***Delivery System Design/ Decision Support*** |  | **0.598** | **0.065** |
|  | Item 4 | 0.593 | 0.073 |
|  | Item 5 | 0.605 | 0.073 |
|  | Item 6 | 0.596 | 0.068 |
| ***Goal setting*** |  | **0.687** | **0.057** |
|  | Item 7 | 0.720 | 0.065 |
|  | Item 8 | 0.736 | 0.050 |
|  | Item 9 | 0.599 | 0.077 |
| ***Problem solving*** |  | **0.673** | **0.054** |
|  | Item 12 | 0.682 | 0.052 |
|  | Item 13 | 0.697 | 0.053 |
|  | Item 14 | 0.698 | 0.056 |
|  | Item 15 | 0.616 | 0.075 |
| ***Follow-up/ Coordination*** |  | **0.704** | **0.061** |
|  | Item 19 | 0.704 | 0.061 |
|  | Item 20 | 0.704 | 0.061 |

Table legend: scalability *H* ≥ 0.50 = strong, 0.49 to 0.40 = moderate, 0.39 to 0.30 = weak, while values of < 0.30 are not considered as unidimensional.

Patient Assessment Chronic Illness Care (PACIC) and its associations with quality of life among Swiss patients with systemic sclerosis: a mixed methods study. Kocher A, Simon M, Dwyer AA, Blatter C, Bogdanovic C, Künzler-Heule P, Villiger PM, Dan D, Distler O, Walker UA, Nicca D. Orphanet Journal of Rare Diseases**.** DOI: 10.1186/s13023-022-02604-2

Corresponding author: Agnes Kocher, Institute of Nursing Science (INS), Department Public Health (DPH), Faculty of Medicine, University of Basel, Switzerland, [agnes.kocher@unibas.ch](mailto:agnes.kocher@unibas.ch)
