# Supplementary material for: Patient Assessment Chronic Illness Care (PACIC) and its associations with quality of life among Swiss patients with systemic sclerosis: a mixed methods study
Source: Orphanet J Rare Dis. 2023 Jan 9;18:7. doi: 10.1186/s13023-022-02604-2 (PMC9828378; doi:10.1186/s13023-022-02604-2)
Supplement: Supplementary file 2 — Additional file 2. Correlation matrix (pearson’s r) of mean PACIC-15 and SScQoL scores. [file 13023_2022_2604_MOESM2_ESM.docx]

**Additional file 2**

***Correlation matrix (pearson’s r) of mean PACIC-15 and SScQoL scores***


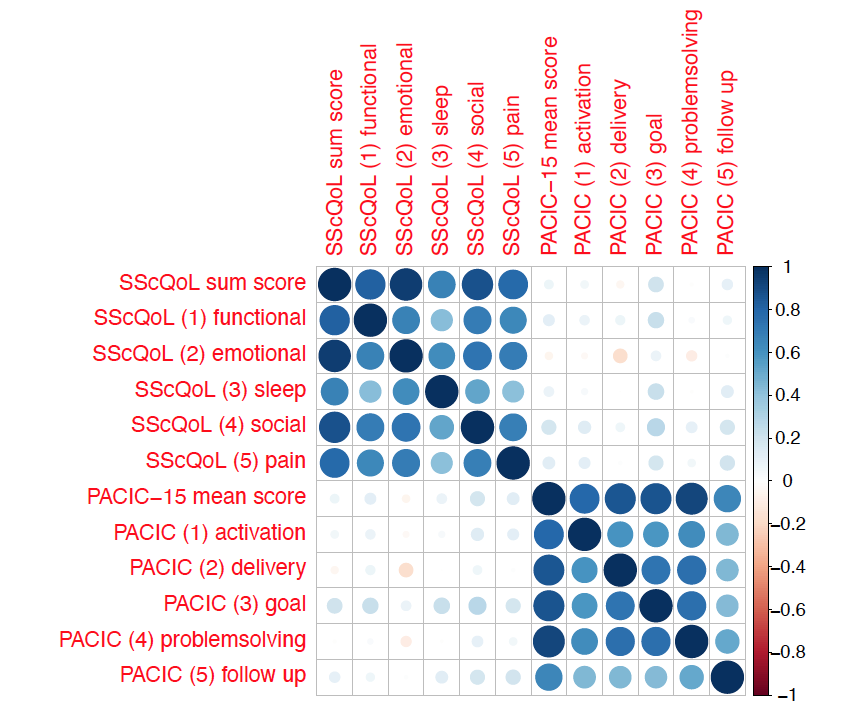


Patient Assessment Chronic Illness Care (PACIC) and its associations with quality of life among Swiss patients with systemic sclerosis: a mixed methods study. Kocher A, Simon M, Dwyer AA, Blatter C, Bogdanovic C, Künzler-Heule P, Villiger PM, Dan D, Distler O, Walker UA, Nicca D. Orphanet Journal of Rare Diseases**.** DOI: 10.1186/s13023-022-02604-2

Corresponding author: Agnes Kocher, Institute of Nursing Science (INS), Department Public Health (DPH), Faculty of Medicine, University of Basel, Switzerland, [agnes.kocher@unibas.ch](mailto:agnes.kocher@unibas.ch)
